# Downregulation of Cinnamyl-Alcohol Dehydrogenase in Switchgrass by RNA Silencing Results in Enhanced Glucose Release after Cellulase Treatment

**DOI:** 10.1371/journal.pone.0016416

**Published:** 2011-01-27

**Authors:** Aaron J. Saathoff, Gautam Sarath, Elaine K. Chow, Bruce S. Dien, Christian M. Tobias

**Affiliations:** 1 Grain, Forage, and Bioenergy Research Unit, United States Department of Agriculture-Agricultural Research Service, Lincoln, Nebraska, United States of America; 2 Bioenergy Research Unit, United States Department of Agriculture-Agricultural Research Service, Peoria, Illinois, United States of America; 3 Genomics and Gene Discovery Research Unit, United States Department of Agriculture-Agricultural Research Service, Albany, California, United States of America; Cinvestav, Mexico

## Abstract

Cinnamyl alcohol dehydrogenase (CAD) catalyzes the last step in monolignol biosynthesis and genetic evidence indicates CAD deficiency in grasses both decreases overall lignin, alters lignin structure and increases enzymatic recovery of sugars. To ascertain the effect of CAD downregulation in switchgrass, RNA mediated silencing of CAD was induced through Agrobacterium mediated transformation of cv. “Alamo” with an inverted repeat construct containing a fragment derived from the coding sequence of *PviCAD2*. The resulting primary transformants accumulated less CAD RNA transcript and protein than control transformants and were demonstrated to be stably transformed with between 1 and 5 copies of the T-DNA. CAD activity against coniferaldehyde, and sinapaldehyde in stems of silenced lines was significantly reduced as was overall lignin and cutin. Glucose release from ground samples pretreated with ammonium hydroxide and digested with cellulases was greater than in control transformants. When stained with the lignin and cutin specific stain phloroglucinol-HCl the staining intensity of one line indicated greater incorporation of hydroxycinnamyl aldehydes in the lignin.

## Introduction

Recalcitrance of biomass to enzymatic digestion reduces the efficiencies of processes that utilize fermentable sugars from lignocellulosic material for the production of ethanol and other biofuels. Though grasses are less recalcitrant than woody perennials, slow digestion rates and requirements for substantial quantities of cell wall degrading enzymes add significant process costs and inefficiencies [1]. Currently several different pretreatment and enzymatic saccharification technologies are undergoing evaluation for the hydrolysis of structural carbohydrates into fermentable sugars. Barriers to cell wall deconstruction include the heterogeneous, cross-linked, nature of the cell wall, the crystalline nature of the cellulose that prevents enzyme access, the variety of covalent and hydrogen bonded linkages that must be cleaved, feedback inhibition of the products of hydrolysis [2], and the presence of enzymatic inhibitors including furans produced by thermochemical breakdown of sugars, phenolic compounds, and carboxylic acids. Much effort has gone into finding optimal processes for feedstock digestion. However, the composition of biomass feedstocks themselves is variable and may also be bioengineered for altered cell wall architecture. Improving biomass quality parameters is a key focus for the development of plants with enhanced conversion properties for use in future biorefineries [3], [4].

Grasses are highly productive and easier to breakdown than woody biomass. Tall, perennial, C4 grasses, such as switchgrass, have shown promise as dedicated energy crops and the costs associated with switchgrass production, transportation, and conversion to biofuels have been studied extensively [5], [6]. These studies indicate that energy return is favorable for this species due in part to its high-yields, low production costs, and adaptability that allows it thrive in marginal areas that are unsuited to food production [7]–[12].

Our current understanding of the structure and biogenesis of the complex cell wall in grasses is limited, but a large number of studies indicate that lignin and related phenolic components of the cell wall limit accessibility of cellulose to degrading enzymes by extensively cross linking cell wall components and are also associated with enzyme and microbial inhibitors produced during some biomass pretreatments [13]–[16]. It is therefore a limiting factor in production of biofuels via enzymatic methods. Lignin is comprised of the monolignol subunits of *p*-coumaryl alcohol, sinapyl alcohol, and coniferyl alcohol that are the products of the a multistep pathway derived from phenylalanine Cinnamyl alcohol dehydrogenase (EC 1.1.1.195) catalyzes the final step in monolignol biosynthesis [17]. These monolignols undergo co-polymerization, possibly through a redox shuttle mechanism [18], within the wall to covalently crosslink one another and give rise to *p*-hydroxyphenyl (H), guaiacyl (G), and syringyl (S) lignin subunits in a racemic mixture of C-C and ether bonds. Genetic approaches to cell wall modification in plants and to understanding the importance of cinnamyl alcohol dehydrogenase in formation of native lignin have demonstrated that its loss of function does not drastically affect fitness. In both monocots and dicots genome-wide characterization indicates that divergence of individual CAD family members predates divergence of major plant lineages [19]–[21]. Some Type I CAD gene knockouts produce a functional modified lignin through the incorporation of cinnamyl aldehyde subunits [22] and perhaps through the overlapping specificities of related gene family members. This process leads in some cases to coloration of CAD deficient lignin. In pine CAD-deficiency leads to orange colored wood, while in sorghum, rice, and maize CAD deficiency produces brownish red internodes, leaf midribs and hulls in rice. More importantly, the modified lignin is more reactive. This has been demonstrated through studies of the pulping properties of CAD deficient wood [23], through rumen digestibility, and through enhanced conversion to ethanol [24]–[26]. In CAD deficient, *bm1* mutants of maize differences in cell wall crosslinking, and a reduction in the amount of esterified *p-*coumarate were observed [27]. Whereas in maize *bm1* lines no difference in overall lignin was reported, in sorghum CAD deficient *bmr-6* mutations cause both a decrease in lignin and an increase in cinnamyl aldehydes [28], [29]. Similarly, in rice the Gold Hull 2 mutant led to a 5–6% reduction in Klason lignin and a reduction in H, G, and S lignin monomers that were presumably replaced with altered cinnamyl aldehydes as suggested by phloroglucinol staining [30]. Overall the agricultural fitness of Type I CAD-mutants was less affected in maize and sorghum relative to mutants in other steps in the monolignol pathway [31]. However in rice a T-DNA insertion into a member of the Type III CAD gene family causes a marked reduction in mechanical strength, plant stature, and production yield which indicated some functional diversification in the CAD gene family and important roles in the biomechanical properties of the cell wall [30], [32].

Transgenic silencing of CAD in grasses has been attempted. A Type I tall fescue CAD was silenced using an antisense RNA strategy and the resulting plants contained 30–50% lower CAD activity, a 15% reduction in klason lignin, increased IVDMD, and less G and S lignin monomers [24]. Silencing in eudicots has generally given similar results where it has been attempted in tobacco, alfalfa, and poplar [33]–[35]. The impact of lignin pathway disruption can have undesirable phenotypes such as reduced yield, sterility, greater drought susceptibility, lodging, and reduced resistance to insect pests and microbial pathogens [31]. Although reports of these consequences in CAD deficient plants are scarce, these traits must be critically evaluated when developing new commercial cultivars targeted for bioenergy. One study indicated Sorghum *bmr-6* mutants show altered colonization by *Fusarium* ssp [36]. In this case CAD deficiency reduced overall lesion length significantly in artificially inoculated peduncles. Maize CAD-deficient *bm1* mutants appear to be earlier flowering than their isogenic counterparts [37]. Two specific CAD inhibitors appeared to reduce the hypersensitive response associated with resistance to stem rust in wheat and powdery mildew in barley [38], [39].

In switchgrass two CAD genes (*PviCAD1* and *PviCAD2*) have been identified and are implicated in lignification based on sequence identity, substrate specificity, and expression pattern [40]. Both genes are members of the Type I family of CADs of which some have demonstrated direct roles in lignification [20], [41]. In this study we employed an RNAi approach to downregulate transcription of the switchgrass CADs most likely to be involved in plant lignification with the goal of improving enzymatic saccharification. We have molecularly characterized a population of switchgrass regenerants transformed with an RNA hairpin generating construct containing an inverted repeat derived from a fragment of the *PviCAD2* gene. Evidence for reduced lignification and enhanced saccharification under bench-scale conditions provide the first evidence for potentially improved conversion characteristics in switchgrass through manipulation of the lignin pathway.

## Results

**Figure 1:**
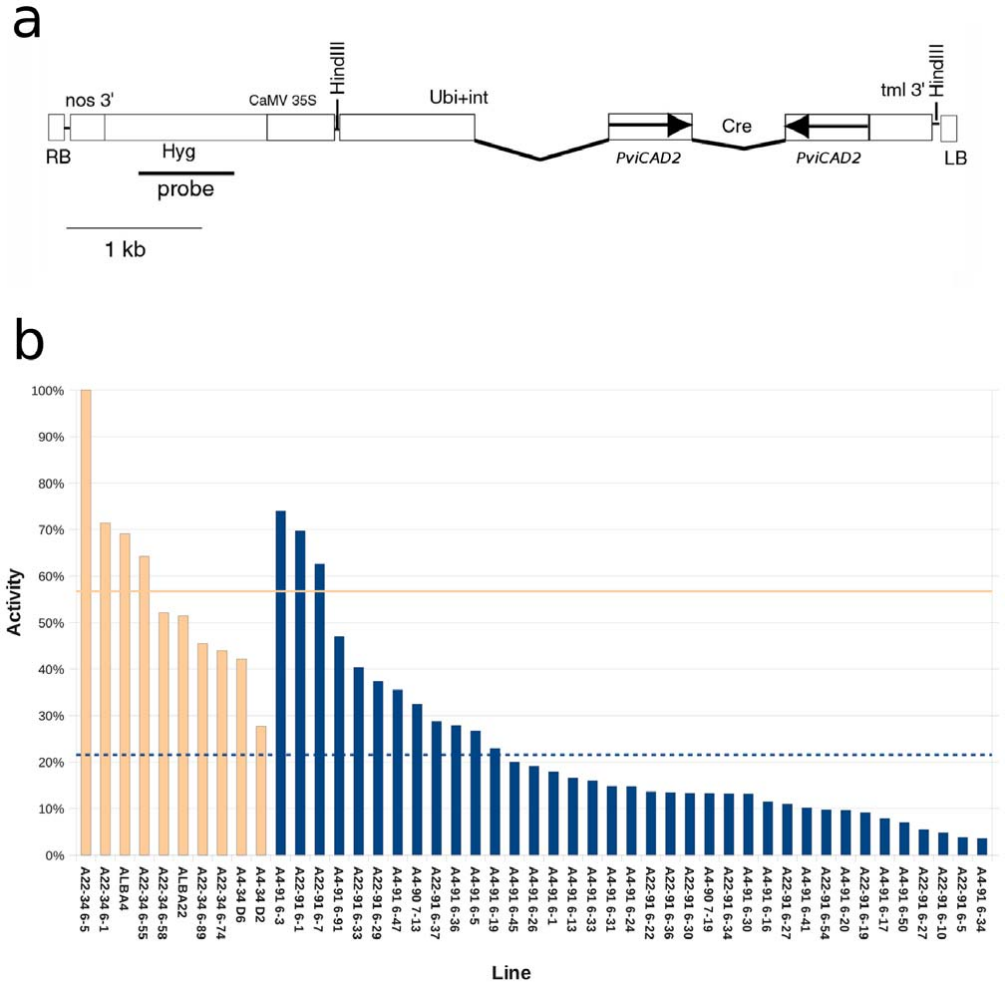
Design of silencing construct used for transformation and screening of regenerants for CAD activity. (**a**) The region between the T-DNA left (LB) and right (RB) regions are shown as open rectangles. Arrows indicate orientation relative to the direction of normal transcription.Abbreviations: Nopaline synthase terminator (nos3′); Maize ubiquitin promoter and 5′ untranslated intron (Ubi+int); Hygromycin *hpt* antibiotic resistance marker (Hyg); Cauliflower Mosaic Virus 35S promoter (CaMV 35S); *A. tumefaciens Tml* 3′ transcriptional terminator (tml 3′); Barley *Cre* intron (Cre); Nucleotides nucleotides 153–727 of *PviCAD2* (*PviCAD2*). A line below the drawing representing 1 kb is included for scale. The region used as a probe for DNA blot-hybridization is shown as a solid line below the drawing. (**b**) Reduction of coniferaldehyde (forward direction) in stem extracts. Activity is expressed as a percentage of highest level. Control untransformed regenerants and vector transformed regenerants are light orange. Individual regenerants transformed with pEC129 are shown as dark blue. Dashed light orange and dark blue lines indicate averages of the control lines and pEC129 regenerants respectively. Lines derived from ALBA22 are indicated with the prefix A22 and lines derived from ALBA4 are indicated with the prefix A4.

Two switchgrass genotypes of the cultivar “Alamo” (ALBA4 and ALBA22) were used for generation of embryogenic callus for the purposes of transformation with *Agrobacterium tumefaciens* containing a control construct and CAD silencing constructs. The control construct (pWBVec8) contained the CaMV35S::hpt::nos3′ selectable marker cassette. The silencing construct (pEC129) was derived from pWBVec8 and contained two copies of a 575 bp fragment of the *PviCAD2* coding region in inverted orientation separated by a spacer region derived from the barley *Cre* intron. Transcription of this repeat was under the control of the maize ubiquitin promoter. The T-DNA region of pEC129 is depicted in Figure 1a. The silencing strategy was expected to reduce expression of both *PviCAD1* and *PviCAD2* genes which share 96% nucleotide sequence identity in this region. A total of 16 and 14 transformed lines from pWBVec8 and pEC129 respectivly were regenerated from ALBA22 that tested positive for the *hpt* gene by PCR while a total of 15 and 44 pWBVec8 and pEC129 transformed lines were regenerated from ALBA4. A subset of these lines was chosen for CAD activity screening by measuring reduction of coniferaldehyde in stem tissue extracts in a colorimetric assay (Figure 1b). The control untransformed, pWBVec8 and putatively silenced lines derived from A22 and A4 showed substantial variation in CAD activity. The change in absorbance at 340 nm per unit protein for ALBA22 and ALBA4 derived lines were expressed as a percentage of the line with the highest activity. In this initial screen A22 and A4 derived lines were not significantly different from one another and were therefore considered as a single population. The average activity for the control extracts expressed as a percentage of maximum were significantly greater than the average activity of the silenced lines and a subset of the individual regenerants were chosen for further analysis based on these results. Five transformants out of 18 lines observed were rated as sterile. Plant height of one of the lines (A4-91 6–24) was significantly less than the others, but significant differences were not observed in growth stage among the lines with normal flowers. Those lines that did not produce flowers were difficult to stage with precision, but did appear to transition from vegetative to reproductive development, undergoing primary and secondary branching of the main axis. No decumbency or lodging was observed.

**Figure 2:**
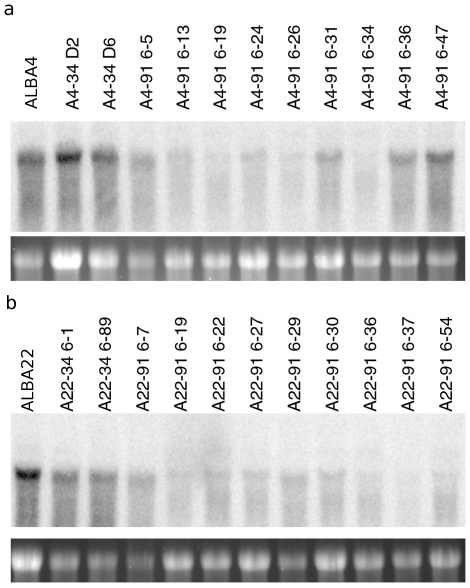
RNA blot-hybridization of lines transformed with CAD silencing construct pEC129. (**a**) Top panel shows autoradiogram of total RNA from individuals derived from line ALBA4 and hybridized with a radiolabelled *PviCAD2* probe derived from the 3′ region of the coding sequence separate from the region used for silencing. Bottom panel is an image of the ethidium bromide stained gel for reference. Labels above the autoradiogram indicate individual identity. The first three individuals on the left are control untransformed (ALBA4) and vector transformed (A4 34-D2 and D6). (**b**) Same as in (a) but with total RNA from lines derived from ALBA22. First three individuals on the left are control untransformed (ALBA22) and vector transformed (A22-34 6-1 and 6–89).

Total RNA was isolated from stem internode tissue of a subset of lines with lower CAD activity as well as untransformed ALBA4 and ALBA22. These RNAs were subjected to blot-hybridizion with a radiolabeled probe corresponding to *PviCAD2* outside of the silencing fragment in the 3′ coding region to quantify endogenous levels of *PviCAD* transcripts likely including both *PviCAD1* and *PviCAD2* as these share 94% nucleotide identity in the probe region. Results are shown in Figures 2a and 2b. Steady state *PviCAD* transcripts in stem tissue were reduced substantially in many of the lines evaluated except for lines A4-91 6–47, A4-91 6-36, and A22-91 6–7. However, two of these (A22-91 6–7 and A4-91 6–47) showed relatively high CAD activity in the initial CAD screen, therefore the CAD activity and RNA blot analysis were consistent with one another. Likewise, the pWBVec8 transformants consistently showed the greatest amount of hybridization to the *PviCAD2* probe.

**Figure 3:**
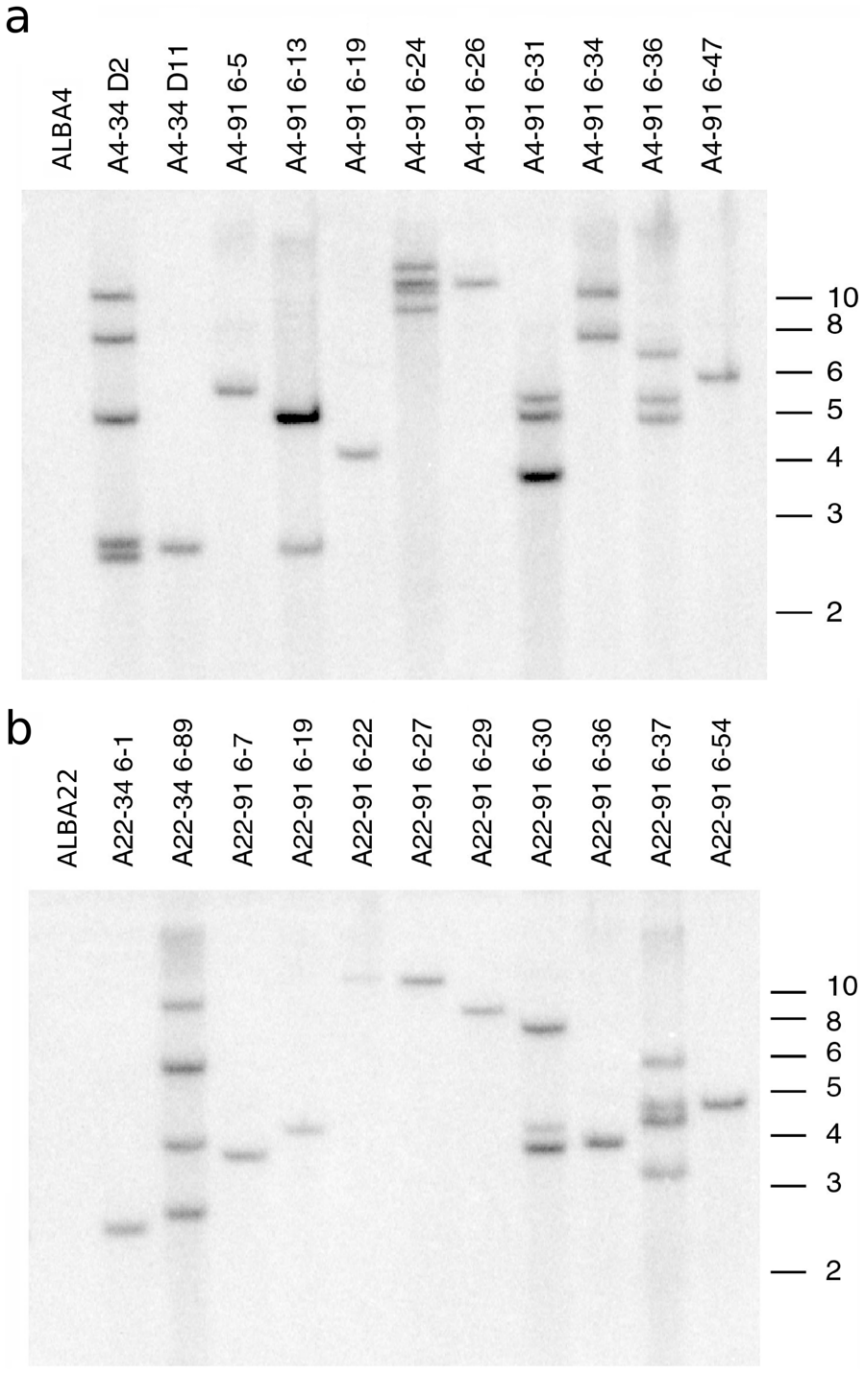
Stable integration and estimated copy number of switchgrass CAD silenced lines. Autoradiogram of DNA blot hybridized with a radiolabelled probe from within the *hpt* coding region (**a**) Untransformed (far left) and pWBVec8-transformed (second and third from left) lines as well as pEC129-transformed individuals derived from ALBA4. (**b**) Same as in (a) but individuals are derived from ALBA22. Untransformed (far left) and pWBVec8-transformed individuals (second and third from left). Size in kilobases are indicated to the right of each panel.

Next we determined if these same lines were stably transformed and determined the transgene copy number. DNA blot hybridization results presented in Figure 3 indicated a total of 13 lines out of the 22 evaluated appeared to contain a single integration of the transgene. The other lines appeared to contain between two and five copies of the transgene based on hybridization signal intensity and number of bands. There was not a strong correlation between copy number and degree of silencing as determined by RNA blot hybridization.

**Figure 4:**
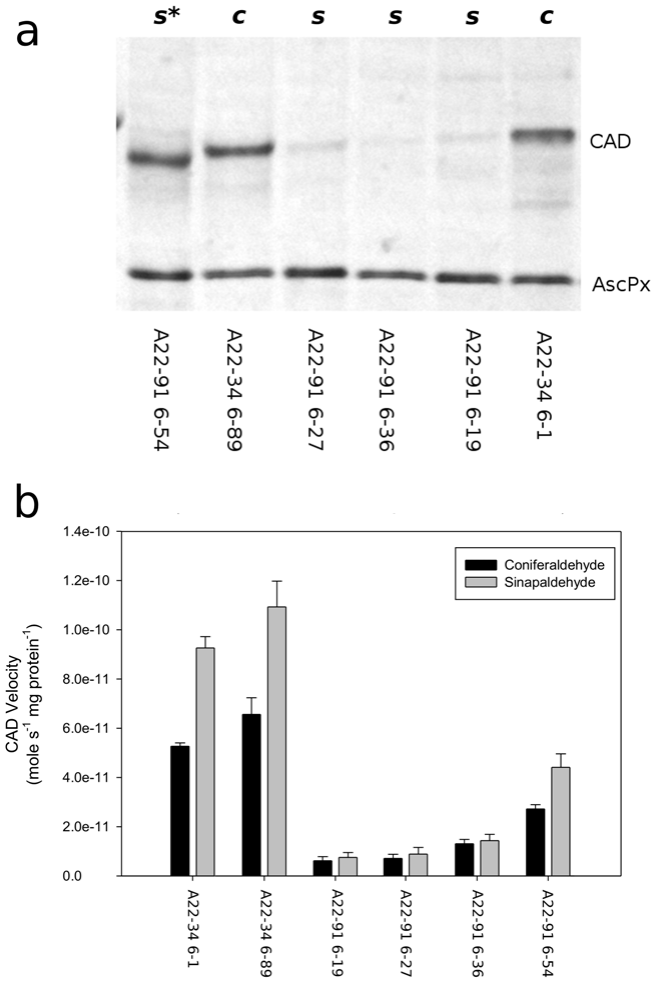
CAD protein expression and enzyme activity in selected CAD silenced individuals. (**a**) Protein immunoblot of silenced individuals (*s*) or control individuals (*c*) indicated above the panel. The positions of the CAD and ascorbate peroxidase (AscPx) proteins are indicated to the right of the panel. An individual producing an immunoreactive protein with altered mobility is indicated by an asterisk. (**b**) Enzyme activity in stem extracts using the substrates coniferaldehyde (black bars) and sinapaldehyde (grey bars). Bars indicate standard error associated with each sample.

Six of the ALBA22 derived lines with apparently single copy T-DNA integrations were subsequently selected and subjected to a more detailed analysis. Total soluble protein was extracted from stem internodes of these six silenced lines and controls and subjected to protein immunoblotting with polyclonal antisera produced against a purified recombinant sorghum BMR-6 (CAD) protein [42] as well as antisera to ascorbate peroxidase (which was used as a loading control). This blot is shown in Figure 4a. Extracts from all the lines showed equivalent reactions to ascorbate peroxidase antisera while three of the silenced lines showed greatly reduced reactivity against the BMR-6 antisera. This indicated that CAD protein levels were reduced in these three lines. One of the pEC129 transformed lines (A22-91 6–54) that was believed to be silenced based on initial screening and RNA blot analysis reacted strongly to the BMR-6 antisera. However, CAD protein mobility appeared to be slightly greater by SDS gel electrophoresis than the control lines indicating the CAD protein itself may be aberrant in this line.

Further enzyme assays were conducted with both coniferaldehyde and sinapaldehyde using more controlled growth, extraction, and measurement conditions to determine the activity within stem internode extracts of these six selected lines and these data are shown in Figure 4b. The CAD activity using both sinapaldehyde and coniferaldehyde as substrates for the four silenced lines, including A22-91 6–54 was substantially reduced relative to the control pWBVec8 transformed lines. The lowest activity was observed in line A22-91 6–19 which was 9.4% and 6.9% as active as A22-34 6–89 against coniferaldehyde and sinapaldehyde respectively, while A22-91 6–54 was 41.5% and 40.4% as active as A22-34 6–89 against coniferaldehyde and sinapaldehyde. All of the extracts were more active with added sinapaldehyde; the maximum rate observed was 1.1×10^−10^ mole sec^−1^ mg protein^−1^ while the maximum rate observed with added coniferaldehyde was 6.6×10^−11^ mole sec^−1^ mg protein^−1^ for the same pWBVec8 transformed line (A22-34 6–89). This finding agrees with previous enzymatic characterization of CAD activity in extracts of switchgrass stems [40].

Ground internode samples of the same six lines were subjected to forage fiber analysis and the results are summarized in Table 1. The six lines had significantly different estimates of the quantities of soluble solids, ash, cellulose, and hemicellulose, but these were not consistently associated with either control or CAD silenced lines. However, significantly lower lignin+cutin values on a percent by weight basis were measured in the four pEC129 transformants relative to the control lines. Line A22-91 6–19 contained 5.37% lignin+cutin which was significantly less than the other pEC129 transformants. This line also had the least activity in stem extracts against coniferaldehyde and sinapaldehyde substrates. On average the four pEC129 lines contained 23% less lignin+cutin than the two control lines. Total caloric value was significantly lower in the pEC129 transformants than in the two pWBVec8 transformants and line A22-91 6–19 again had the lowest total caloric value at 4352 cal g^−1^ dry wt. On average the four silenced-lines caloric value was reduced by 1.6% relative to the two pWBVec8 transformants.

**Table 1:** Fiber analysis of CAD silenced transformants.

Vector	Line	lignin + cutin^1^	cellulose	hemicellulose	cell solubles	Ash	cal g^−1^ dry wt
pWBVec8	A22-34 6-1	8.04±0.1a^2^	39.67±0.45 ab	33.58±0.57 a	18.72±0.56 d	2.41±0.03 c	4461±5 a
	A22-34 6-89	7.84±0.05 a	38.47±0.38 b	32.22±0.57 ab	21.46±0.22 c	2.45±0.01 c	4445±4 a
pEC129	A22-91 6-19	5.37±0.06 c	38.09±0.35 b	33.09±0.39 ab	23.44±0.59 b	3.14±0.06 a	4352±7 c
	A22-91 6-27	6.61±0.16 b	40.40±0.36 a	32.04±0.19 ab	20.96±0.47 c	3.28±0.04 a	4388±12 bc
	A22-91 6-36	6.46±0.06 b	38.89±0.47 ab	32.03±0.34 ab	22.62±0.16 bc	3.19±0.04 a	4403±5 b
	A22-91 6-54	6.30±0.08 b	34.81±0.27 c	31.51±0.14 b	27.38±0.22 a	2.88±0.06 b	4390±9 b

1 Values for lignin+cutin, cellulose, hemicellulose, and cell solubles are reported as percent dry wt.

2 Samples with different letters are significantly different at the 0.05 level by Tukey's HSD.

**Figure 5:**
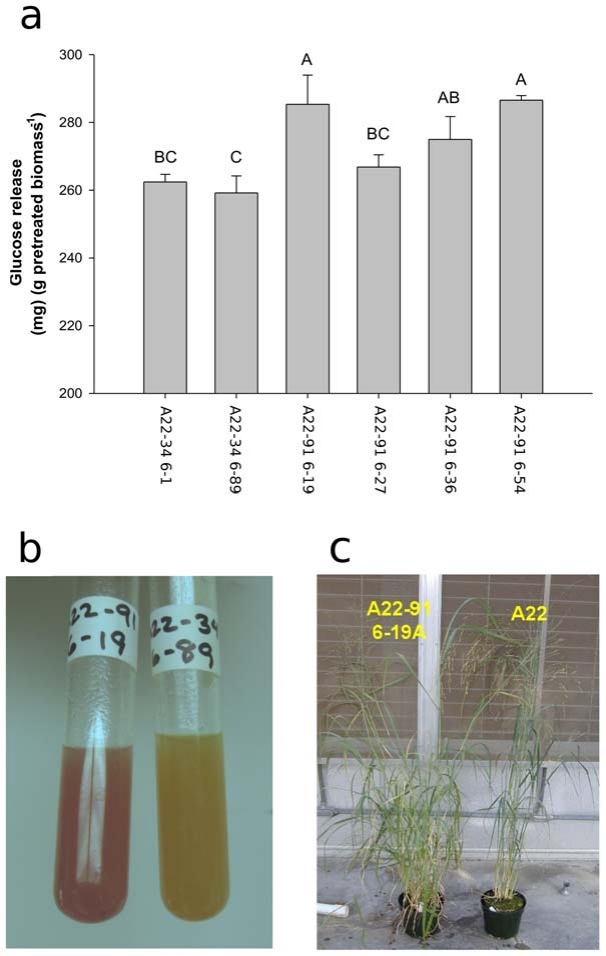
Altered susceptibility to enzymes and composition of the cell wall in CAD silenced lines. (**a**) Glucose release from pretreated pWBVec8 transformed lines A22-34 6-1 and A22-34 6–89 on the left and selected pEC129 transformants (right) expressed as mg glucose per gram dried sample as a result of treatment with cellulclast and β-glucosidase. Error bars indicate standard error of the mean (n = 3). Samples with the same letter located above the bars are not significantly different. Results shown are the mean of three cellulose digests on separate pretreatments. (**b**) Photograph of phloroglucinol stained ground cell wall extracts from CAD-silenced line A22-91 6–19 (left) and A22-34 6–89 (right). (**c**) Appearance of A22-91 6–19 (left) relative to control untransformed line A22 (right) grown in the greenhouse.

The effect of lowering CAD activity on cell wall digestibility was assessed by enzymatic saccharification with cell wall samples pretreated with ammonium hydroxide and then placed in a commercial cocktail of cellulases and β-glucoside. Glucose yields are shown in Figure 5a. These data indicated that all of the samples from the pEC129 transformed lines were more susceptible to saccharification after pretreatment. In two pEC129 lines the total amount of glucose released was significantly different (α = 0.05) than either of the pWBVec8 transformed lines. There was no significant correlation between glucose release and cellulose content from the fiber analysis. To determine if the structure of the lignin may be altered in the silenced individuals, we stained ground cell wall material from midribs of leaf blades with phloroglucinol-HCl and immediately photographed the results. Immediately upon addition of phloroglucinol the silenced line A22-91 6–19 appeared bright red while the control line A22-34 6–89 produced a positive reaction that was less intense. These results are shown in Figure 5b. Coloration differences became less distinct with time and differences in phloroglucinol reactivity were not seen in the other three silenced lines. The different staining intensity strongly suggested A22-91 6–19 contained a modified lignin. The appearance of A22-91 6–19 compared to ALBA22 grown under greenhouse conditions is shown in Figure 5c.

## Discussion

Cinnamyl alcohol dehydrogenases have been found to control both the quantity and composition of lignin in the cell wall. In grasses CAD mutants have been isolated in rice, sorghum, and maize that produce altered lignin and lead to the brown-midrib phenotype as well as discolored awns and internodes in some species. Biotechnological approaches to increasing forage digestibility by the silencing of Type II CADs has been successful in Tall Fescue [24] which encouraged us to pursue this approach for switchgrass even though there were likely to be at least two members of the gene family [40]. In this study we used strong, ubiquitin-promoter driven expression of an inverted repeat, RNA stem-loop to induce silencing of CAD in switchgrass. One of the components of this strategy was to eliminate potential sources of variation caused by individual genetic makeup by using only two genotypes for culturing and transformation. These were derived from the “Alamo” cultivar, which has previously been demonstrated to have better transformation and regeneration efficiency than other named cultivars and is a high yielding, lowland ecotype. This strategy may be useful when attempting controlled transformation experiments in switchgrass as natural phenotypic variation would otherwise reduce the likelihood of detecting small predicted differences. The strategy thus reduces the need to assess common phenotypic variation during analysis. A different strategy was chosen by Somleva *et al.* [43] to produce PHA plastic in switchgrass. In this case since there is no variation for this trait and maximal PHA production was the goal, transformation of a large number of genotypes from embrogenic callus derived from mature caryopses was justified.

For the purposes of identifying CAD-silenced lines for further analysis we visually screened leaf blades and sheaths for reddish brown discoloration and also stained sectioned leaves and stems with phloroglucinol-HCl. However no changes in coloration or phloroglucinol staining were detected as we had predicted would be the case based on previous results with sorghum *bmr6* mutants [28], [24]. Absence of a brown-midrib phenotype also is true for CAD-silenced tall fescue [24]. Therefore we looked for other indicators during screening particularly enzyme activity. In rice Gold Hull 2 (*gh2*) mutants the bmr phenotype is absent in the midrib, but orange discoloration appears in the hull and internode [30]. A different member of the CAD gene family in rice that is knocked out by a T-DNA insertion causes the *flexible culm 1* mutation. The gene that was knocked out belonged to a Type III CAD family member [32]. Incorporation of novel monolignol monomers via substitution for coniferyl, *p*-coumaryl, and sinapyl alcohols is believed to be the cause of this discoloration and also be responsible for enhanced phloroglucinol staining. This enhanced phloroglucinol staining was therefore expected. However, it was only clearly evident in silenced line A22-91 6–19. This line appeared to have the greatest reduction in CAD activity and also was the most susceptible to enzymatic digestion indicating that CAD activity may need to drop below a minimum threshold in switchgrass before it becomes rate limiting as has been concluded in other species [44].

Measuring CAD activity in extracts with added coniferaldehyde was used effectively to screen larger numbers of CAD PCR positive regenerants and those with reduced activity were used for further analysis. This approach appeared to be successful, in spite of the large variations in CAD activity detected within the control pWBVec8, and untransformed lines. Variation within the pEC129 transformed lines was expected due to variations in T-DNA copy number that are typical with Agrobacterium mediated transformation and due to genome composition surrounding the insertion site. The four pEC129 transformed lines ultimately chosen for detailed analysis were all from the lower end of the distribution of CAD activity of the group as a whole. The CAD activity and protein from silenced line A22-91 6–27 was suppressed to a similar level as A22-91 6–19. However, it did not show increases in digestibility or decreases in caloric value and lignin+cutin to the same extent as A22-91 6–19. The reasons for this are unclear; as lignin content is under both environmental and developmental control it may be that unforeseen variation in growth conditions or sampling effects influenced the results.

DNA blot analysis of regenerants was used to estimate T-DNA copy number. We observed that 13 out of 22 (55%) of regenerants produced a single band and were therefore likely to have one integrated T-DNA. This percentage is higher than the ranges reported for Agrobacterium mediated transformation of some other species [45], [46]. Higher T-DNA copy number is correlated with silencing of both selectable marker and transgenes and is also difficult to use for breeding purposes, therefore these results were encouraging.

Enzymatic saccharification activity was significantly higher in A22-91 6–19 using commercial cellulases. This effect may be the result of either or both increased substrate availability or decreased cellulytic enzyme inhibition. Little data is currently available on the direct effects of lignin modification particularly with grasses on enzymatic susceptibility which is important for bioenergy applications. The best data available thus far in sorghum bmr6 mutant alleles shows increases in digestibility of 7% and 20% relative to the specific genetic background for each allele due to either differences in penetrance or genetic background affects [47]–[49]. Our results are the first to indicate improved enzymatic susceptibility and improved glucose yields for any form of cellulytic digestion with switchgrass engineered to produce a modified lignin and the first to demonstrate that lignin-modified grasses pretreated under alkaline conditions also release more glucose. The results indicate that this increase in glucose release was modest (2–11%), but proves the potential benefits of the approach in switchgrass. Further analysis of ethanol production under different pretreatment and fermentation procedures should give better estimates of yield and energy return relative to available switchgrass cultivars.

## Materials and Methods

### Cloning and Plant Transformation

PviCAD2 sequences were amplified from a plasmid clone containing the entire coding sequence of PviCAD2 (*PviCAD*2, GenBank accession no. GU045612) with the primers 5′-CTACGTATTAATTAACACCCACCAGGCCAAGA-3′ and 5′-GACTAGTGGCGCGCCGGCCGCCGCCATGGCCTCCG-3′ that incorporated *Pac*I/*Sna*BI and *Asc*I/*Spe*I cloning sites respectively. The cloned PCR product was sequence verified and sequentially cloned into the corresponding sites of pCLARICEIII [50] to place 575 bp of the *PviCAD2* coding sequence from nucleotides 153–727 relative to GU045612 in opposite orientations separated by a spacer sequence derived from the Barley *Cre* intron. The plasmid vector pCLARICEIII is designed to transcribe sequences under control of the maize *Ubi* promoter and intron with a *Tml* 3′ transcriptional terminator derived from *Agrobacterium tumefaciens*. The resulting vector was digested with *Not*I and inserted into the binary vector pWBvec8 [51] to generate pEC129 that contained the hygromycin selectable marker under the control of the CaMV35S promoter oriented so that the UBI and CaMV35S promoters were adjacent to one another and transcribed in opposite orientations with the *Tml* 3′ end adjacent to the left border. Transformations were carried out via Agrobacterium co-cultivation of embryogenic callus using *A. tumefaciens* strain AGL1[52]. Callus was derived from two genotypes ALBA4 [53] and ALBA22 originally obtained from a screen for regenerability from the switchgrass cultivar “Alamo”. Transformation procedures were identical to those of Somleva *et al.*, 2002 [54]. Transformants were grown routinely in a greenhouse at the Western Regional Research Center, under a 16 h 26–30°C day/8 h 22–26°C night growth regimen, using supplemental lighting from halide lamps (200 mol photons m^−2^ s^−1^) in Sunshine Mix #1 (Sun Gro Horticulture, Vancouver, CA). Plants were watered weekly with a nutrient solution containing 200 ppm N and with tap water as needed otherwise. Replicated greenhouse experiments were staged using the method of Moore *et al.* 1991 [55] and phenotypic data was tested for significance using Tukey's HSD test.

### Nucleic Acid Blot Analysis

Genomic DNA from individual regenerants was isolated using a CTAB extraction procedure and digested with *Hind*III. A portion of the digest (∼100 ng) was checked by electrophoresis to verify complete digestion and to quantify. After quantitation 10 µg of digested DNA was separated on a 0.8% agarose gel overnight at 0.10 V/cm, subjected to partial acid hydrolysis and transferred to positively charged nylon membranes (Roche, Indianapolis IN). The membranes were incubated in the presence of a ^32^P [α-dCTP] radiolabelled probe corresponding to 558 bp within the hygromycin gene overnight at 65°C. The membranes were then washed twice in 1 x SSC 0.1% (w/v) SDS at 65°C and once in 0.2xSSC, 0.1% (w/v) SDS at 65°C for 30 minutes prior to exposure to a phosphor screen and subsequent imaging using a Molecular Dynamics Storm 820.

Total RNA was isolated from stem internodes using trizol reagent and subjected to denaturing agarose gel electrophoresis in the presence of formaldehyde using MOPS buffer. Gels were run at 80 V constant voltage in the presence of RNA size standards (Ambion, Austin TX) in the presence of ethidium bromide and photographed under ultraviolet irradiation. The RNA was transferred to nylon membrane and hybridized with a radiolabeled PCR probe corresponding to the C-terminal 122 amino acids of PviCAD2 outside of the region used for silencing which was amplified with the primers sequences 5′-GAATTCAGTTGGCCGGCGCCC-3′ and 5′-CTCGCTGGACTACATCATCGACACGG-3′. Blots were hybridized with probe DNA in the presence of stDNA in phosphate buffer overnight at 65°C and washed twice for 10 m in 0.1xSSC, 0.1% (w/v) SDS. Membranes were then imaged as for DNA blots.

### Protein Blot Analysis

For one-dimensional SDS-PAGE, approximately 300 mg of ground internode materials were extracted as described earlier [42] and proteins were separated on 12% polyacrylamide gels [56]. Proteins separated by SDS-PAGE were blotted to nitrocellulose membranes and probed with polyclonal antibodies raised to CAD as described earlier [54].

### Pretreatment/Enzymatic Saccharification

Samples were treated at 15% w/w solids in 8.0% w/v ammonium hydroxide solution at 180°C for 20 min. The mixture was heated in 316 stainless steel tube reactors (20 ml capacity), which were placed in a fluidized sand bath (Model 01187-00 bath and 01190-72 temperature controller, Cole-Parmer, Vernon Hills, IL). The reaction time was based upon the internal reactor temperature, which was monitored using a thermocouple probe inserted into one of the reactors. Following the reaction, the reactors were quickly cooled in a water bath. After cooling, samples were air dried at 25°C for 48 h to remove ammonia.

Enzymatic saccharifications were performed on the ammonia hydroxide pretreated biomass by generally following the published procedure in NREL technical report NREL/TP-510-42629 [57]. Moisture content of the pretreated material was first determined by placing subsamples in drying oven (Lindberg/Blue M Model M01490A-1, General Signal Corp., Asheville, NC) overnight at 105°C. For the cellulase digests, pretreated biomass was placed into a 15 mL tube (Becton Dickinson & Co., Franklin Lakes, NJ) and weighed to ensure each tube contained approximately 0.20 g of material. Deionized water, sodium citrate buffer (0.1 M, pH 4.8), tetracycline, and cyclohexamide were added according to [57]. A cellulase solution (Cellulclast 1.5L, Novozymes, Inc., Franklinton, NC) and β-glucosidase solution (Novo 188, Novozymes, Inc., Franklinton, NC) were added to final amounts of 60 FPU/g cellulose and 64 pNPGU/g cellulose, respectively. Tubes were tightly capped, placed on a tube rotor in a 50°C oven (Hybaid HBSNSR110, Thermo Fisher Scientific, Waltham, MA), and incubated for 116 hours. After incubation, tubes were centrifuged at 3,000 RPM (Beckman GS-6, Beckman Coulter Inc., Brea, CA) for 10 min and 5 mL of supernatant was syringe filtered through a 0.2 µm nylon filter. The filtered solution was stored at −20°C and analyzed for glucose content the next day using ion chromatography (Dionex ICS-3000, Dionex Corp., Sunnyvale, CA).

### Enzyme assays

For each plant, independent extracts were performed in duplicate. Stem internodes were ground to a powder using dry ice and a coffee grinder and stored at −80°C prior to use in CAD assays. Approximately 0.3 g of the frozen material was placed in a 2.0 mL tube with 1.2 mL 100 mM Tris-Cl, pH 7.5 containing 5% (v/v) ethylene glycol and 5 mM DTT with a protease inhibitor cocktail (#P9599, Sigma-Aldrich Co., St. Louis, MO) added immediately prior to use. Samples were placed on ice and sonicated using a Branson Digital Sonifier 450 (Branson Ultrasonic Corp., Danbury, CT) at 20 W 3 times with a 15 s pulse with a 30 s cool down in an ice-ethanol bath between each pulse. Tubes were then centrifuged for 15 min at 13,000 RPM and 4°C. Supernatant was placed into new 1.5 mL tubes on ice and assayed for CAD activity shortly thereafter. Protein content of the extracts was determined using a colorimetric assay (Pierce 660 nm Protein Assay, Pierce Biotechnology, Rockford, IL) and lysozyme was used as a protein standard.

Enzyme activity on each substrate was measured using reaction conditions similar to those previously published [58], [59] with adaptations for use with a microplate reader [42]. Forward reactions (aldehyde conversion to alcohol) used 100 mM MES, pH 6.5, 200 µM NADPH, and 100 µM of the appropriate substrate.

Absorbance was read on a 96-well microplate reader (BioTek Synergy HT, BioTek Instruments, Winooski, VT) at 340 nm. In the case of sinapaldehyde, 420 nm was used. To reduce variability, assays were conducted in quadruplicate and each well was thoroughly mixed by repeated up-and-down pipetting prior to insertion into the plate reader. Total time between extract addition into 4 wells and placement into the plate reader was monitored and kept constant at 35 s. To further minimize any effects of pipetting errors between replicates, the buffer, cofactor, substrate, and water were mixed together prior to placement in microplate wells and subsequent addition of extract, which had the effect of blocking on these variables across concentrations and replicates.

Data from the plate reader was analyzed using SAS for Windows 9.1 (SAS Institute, Inc., Cary, NC). The method of linear least squares was used to fit a simple two parameter model to the optical density data. The first 60–90 s of optical density data was used to generate parameter estimates. Calibration curves using coniferyl and sinapyl aldehydes were generated at both pH 6.5 and pH 8.8 in order to relate optical density to aldehyde concentration. For determining forward reaction rates, data at 340 nm was used since it represented the absorbance maximum of both the aldehyde and NADPH at pH 6.5. In this case, extinction coefficients for both the aldehyde of interest and NADPH were used in calculating the relative contribution of each to the 340 nm signal.

### Fiber Analysis

Dried and ground stem internodes were analyzed for cell wall components using a detergent digestion protocol as described by Vogel *et al.*, (1999) [60]. Triplicate samples were placed in pre-weighed bags and processed by the fiber extraction protocol [60], and the ANKOM ADL procedure (ANKOM Technology-9/99, Method for Determining Acid Detergent Lignin (ADL); ANKOM Technology Corp., Fairport, NY, USA). These procedures estimate neutral detergent fiber (NDF), acid detergent fiber (ADF) acid detergent lignin (ADL). A final combustion step determined ash concentration. The relative percentage of cell wall components, namely cell-solubles, hemicellulose, cellulose and lignin, were calculated as in Sarath *et al.*2007 [61] using component concentrations expressed on a dry weight basis. On an oven dry weight basis (g kg^−1^), cell solubles = sample weight- NDF; hemicellulose = NDF-ADF; cellulose = ADF-ADL; lignin = ADL-ash [62]. Samples were combusted in a bomb calorimeter to determine total caloric value.
